# Seven-Year Follow-Up of Peutz-Jeghers Syndrome

**DOI:** 10.1155/2016/6052181

**Published:** 2016-04-18

**Authors:** Hamid Reza Mozaffari, Fetemeh Rezaei, Roohollah Sharifi, S. Ghasem Mirbahari

**Affiliations:** ^1^Department of Oral Medicine, School of Dentistry, Kermanshah University of Medical Sciences, Kermanshah, Iran; ^2^Department of Endodontics, School of Dentistry, Kermanshah University of Medical Sciences, Kermanshah, Iran; ^3^Department of Pathology, School of Medicine, Kermanshah University of Medical Sciences, Kermanshah, Iran

## Abstract

One of the clinicopathological criteria for diagnosing Peutz-Jeghers syndrome (PJS) is mucocutaneous pigmentation. We present a 57-year-old Iranian female patient with diffuse pigmentation in buccal and labial mucosa. The first colonoscopy revealed one 0.5 cm rectal polyp. However surveillance colonoscopies over a 7-year polyp showed over 100 colorectal polyps.

## 1. Introduction

Peutz-Jeghers syndrome (PJS) is a rare inherited autosomal dominant disease with an incidence of 1 in 12–30,000 live births, characterized by mucocutaneous pigmentation and multiple hamartomatous polyps in the gastrointestinal tract. PJS patients have a marked increase of risk of developing cancer [1].

A germline mutation in* STK11*, a tumour suppressor gene localized to chromosome 19p13.2–13.3, is an underlying abnormality [2].

The clinicopathological criteria of World Health Organisation (WHO) for diagnosing PJS are as follows:

1. Three or more polyps, with histological features of PJS.
2. A family history of PJS with any number of polyps.
3. A family history of PJS with characteristic mucocutaneous pigmentation.
4. Characteristic mucocutaneous pigmentation with any number of polyps [3].

To improve the understanding, diagnosis, and treatment of PJS, we now report a case of PJS.

## 2. Case Present

A 57-year-old Iranian female patient was referred to us (oral medicine specialist), with a chief complaint of pigmentation in oral cavity for more than 15 years.

**Figure 1:**
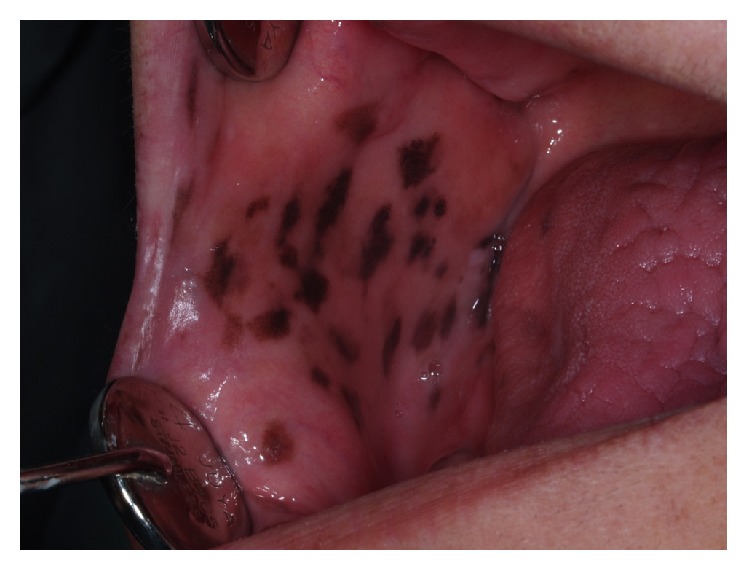
Mucocutaneous pigmentation on the buccal mucosa.

The patient had scattered dark brown to black macules, most of them measuring <0.5 cm on the buccal and labial mucosa. These pigmentations were asymptomatic and well circumscribed and did not fade under pressure (Figure 1).

There were not any similar macules on the tongue, lips, perioral skin, nostril, hands, and feet. She was previously healthy and took no regular medications. She had no abdominal pain, nausea, vomiting, diarrhea, or history of changing in bowel habits or any significant loss of weight or appetite. In family survey, just her brother had PJS with perioral pigmentation and intestinal polyps compatible with Peutz-Jeghers polyp on histology and without any signs and symptoms of GI diseases, but unfortunately he died in car accident many years ago. Her children (two daughters and one boy) do not have any sign or symptom of this syndrome.

**Figure 2:**
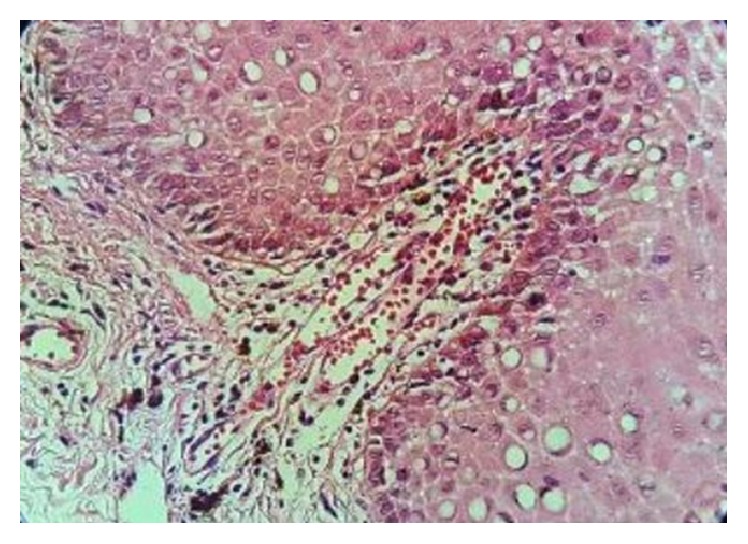
Biopsy of pigmentation on buccal mucosa indicates increased basilar melanin with melanin incontinence into the submucosa (H&E staining).

Biopsy of pigmentation on buccal mucosa was performed. Histopathologically there is evidence of increased basilar melanin with melanin incontinence into the submucosa (Figure 2).

The patient with primary diagnosis of PJS was referred to gastroenterologists for more diagnostic assessments.

In the first colonoscopy, there was only one polyp in the rectum measuring 0.5 cm in diameter, without any features of invasions or intussusceptions.

**Figure 3:**
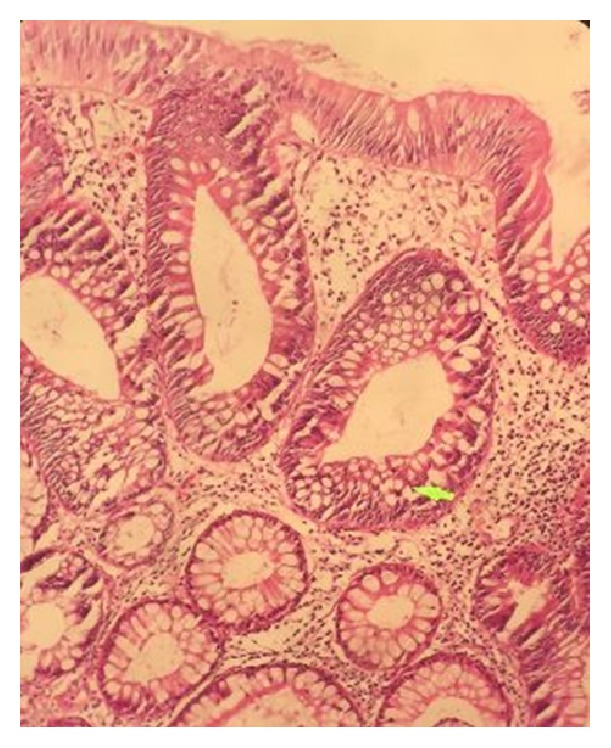
Pathological examination of the polyps confirmed adenomatous and hamartomatous polyps (tubular type), with low grade dysplasia (H&E staining).

In the past 7 years the patient was examined for oral pigmentations and intestinal polyps every year. We did not observe any significant change in oral pigmentations during these years. She also did not have any sign or symptom of GI diseases except mild constipation, but endoscopy of GI showed the increase of the number of polyps every year. In the last colonoscopy there were more than 100 polyps with the size of <1 cm in the large bowel and rectum; no features of invasion and intussusceptions were noticed. Pathological examination of the polyps confirmed adenomatous and hamartomatous polyps (tubular type), with low grade dysplasia (Figure 3).

## 3. Discussion

In 1921, Dutch physician Peutz noted the combination of gastrointestinal polyps and mucocutaneous melanotic spots in three young children [4].

The presented case in this report was of particular interest since there are few reported cases of PJS with long-term follow-up [5]. We were able to visit the patient annually and trace her medical findings. Another important issue in this report was a different course of the disease. Her pigmentation showed little change but there was a dramatic increase in the development of colorectal polyps over 7 years.

Male-to-female ratio of PJS is 1 : 1. The average age at the time of diagnosis is 23 years in men and 26 years in women [6], but our patient was in sixth decade of her life.

Although most of pigmentations of PJS are located around of mouth cheek, nostril, periorbital, ears, and overall orifices of body [3], these pigmentations of our patient were located intraorally in buccal and labial mucosa. Also Mozaffar et al. reported a PJS without mucocutaneous pigmentation [6]. So PJS may have a variable spectrum of manifestations in mucocutaneous pigmentation. The probable explanation is novel mutations in contributing genes [7].

Hamartomatous polyps commonly involve the jejunum and patients usually have complained of GI diseases like abdominal pain, diarrhea or bleeding, and bowel obstruction including intussusception [8]. In our patient the colon was the most common site of these polyps and she did not have any sign and symptom of GI disturbance even after 7 years (with 100 polyps). Also Matini reported three cases of PJS without clinical symptoms like abdominal pain and acute or chronic rectal blood loss and with no evidence of gastrointestinal or extragastrointestinal malignancy during 14 years of follow-up [5].

The treatment for mucocutaneous pigmentations of PJS was usually considered unnecessary; but in some patients, the appearance of brown discoloration, especially on the skin, can be bothersome, so laser therapy can be a choice treatment [9]. The pigmentations of our patient were intraoral and the face skin was not involved so we did not treat them.

The opinions for treatment of polyps are different. Some consider these polyps as premalignant lesions, which should be paid considerable attention and removed by surgery or endoscopy. However, others found out that rate of cancer in these polyps is very low, so periodic endoscopic surveillance is enough [5, 10, 11]. As a limitation in this case report, there was not mutation analysis to find out a defect in the* STK11* gene is responsible for the PJS phenotype in this patient.

In the case we reported that our patient even with diffuse gastrointestinal polyposis does not have any complaint of abdomen pain, bloody stool, or other symptoms. So no particular medication or surgery was administrated while in periodic follow-up.

In conclusion clinical manifestations of PJS may be varied and the clinical course of this disease is not predictable.

## 4. Conclusion

In conclusion, it is recommended that any patient presenting with multiple pigmentation or incomprehensible discoloration in oral cavity should be investigated for systemic diseases like Peutz-Jeghers syndrome.
